# Role of radiotherapy in melanoma management

**DOI:** 10.2478/v10019-010-0008-x

**Published:** 2010-03-18

**Authors:** Primoz Strojan

**Affiliations:** Department of Radiation Oncology, Institute of Oncology Ljubljana, Ljubljana, Slovenia

**Keywords:** melanoma, radiobiology, radiotherapy, fractionation, indications, toxicity

## Abstract

**Background:**

In melanoma, radiotherapy has generally been considered as a palliative treatment option indicated only for advanced cases or disseminated disease. In the 70s of the previous century, the technological advances in radiotherapy, linked to rapid development of computer sciences, resulted in restored interest for radiotherapy in melanoma management. Although a fundamental lack of well designed prospective and/or randomized clinical trials critically influenced the integration of radiotherapy into treatment strategies in melanoma, radiotherapy was recently recognized as an indispensable part in the multidisciplinary management of patients with melanoma. Altogether, approximately 23% of melanoma patients should receive at least one course of radiotherapy during the course of the disease. In this review, radiobiological properties of melanoma that govern the decisions for the fractionation patterns used in the treatment of this disease are described. Moreover, the indications for irradiation and the results of pertinent clinical studies from the literature, creating a rationale for the use of radiotherapy in the management of this disease, are reviewed and a brief description of radiotherapy techniques is given.

**Conclusions:**

Basic treatment modality in melanoma is surgery. However, whenever surgery is not radical or there are adverse prognostic factors identified on histopathological examination of resected tissue specimen, it needs to be supplemented. Also, in patients with unresectable disease or in those not being suitable for major surgery or who refuse proposed surgical intervention, other effective mode(s) of therapy need to be implemented. From this perspective, supported by clinical experiences and literature results, radiotherapy is a valuable option: it is effective and safe, in curative and palliative setting.

## Introduction

Changes in human behavior, particularly those related to sun exposure and global environmental alterations have contributed to an observed increase in the incidence of cutaneous melanoma in Europe since the 1950s.^1^ In Slovenia with the population of two million, the melanoma incidence doubled during the last decade, being 10.2 per 100.000 inhabitants in 1997 and 19.6 in 2006.^2^,^3^ As melanoma is a significant health burden, its management was continuously in focus of extensive laboratory and clinical research.

Surgery is basic and the most effective treatment modality for melanoma, whereas radiotherapy, one of the corn stones of anti-cancer management, has been evolving steadily and, during the time, taking over greater role in the management of this disease. It has long been negatively marked by the lack of well designed prospective and/or randomized clinical trials which finally gave more credit to lucid observations of clinicians dealing with the disease.^4^

First experiences of radiation oncologists with melanoma were marked with technologically inferior irradiation devices and the label of tumor as radioresistant, which originated from categorization of tumor radiosensitivity by histological type introduced in 1930s.^5^ Consequently, radiotherapy was generally considered as a palliative treatment option indicated only for advanced cases or disseminated disease.

In the 70s of previous century, the interest for radiotherapy in melanoma management was restored. During the decades, new knowledge on radiobiological characteristics of melanoma cells as well as more favorable clinical experiences were obtained. Furthermore, modern radiotherapy devices, including treatment planning systems, appeared on the market, allowing more sophisticated treatment planning and accurate targeting. These novelties contributed to a change in perception of clinicians confronted with this disease, which directly contradicted to long standing belief that melanoma is radioresistant tumor.

Nowadays, RT is recognized as the most effective non-surgical mode of locoregional therapy of melanoma and is an integral part of the multidisciplinary management, thus providing a valuable input to the best treatment of melanoma. According to Delaney *et al.*, the recommended proportion of all patients with melanoma who, according to the best available evidence, should receive at least one course of radiotherapy is 23%.^6^

## Clinical radiobiology of melanoma and fractionation pattern

Response of melanoma to irradiation depends on tumor volume, radiotherapy dose and fraction size. From preclinical studies as well as clinical observations, an abundance of evidences emerge confirming a positive relationship between response to irradiation and radiotherapy dose corrected for tumor volume, *i.e.* the number of clonogenic cells that need to be sterilized. Within the timeframe of the schedules used, the overall treatment time showed no effect on response rate.^7^,^8^

**FIGURE 1:**
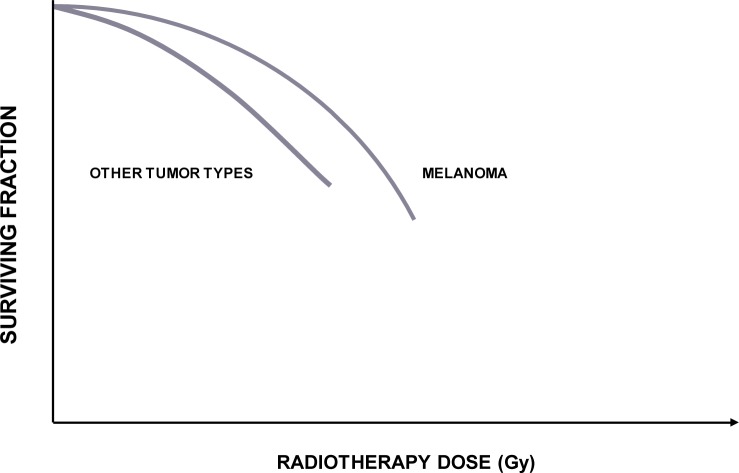
Dose-response curve for melanoma cells. High intrinsic capacity of melanoma cells for repair of sublethal DNA damages caused by irradiation is graphically presented by a distinctly broad shoulder in the low-dose portion of the logarithmic cell survival curve. Accordingly, the ability of melanoma cell to overcome sublethal DNA injuries suggests increased sensitivity to large doses per fraction (hypofractionation).

From radiobiological perspective, the most intriguing is the observation that melanomas have a wide range of sensitivities to ionizing irradiation.^9^–^13^ The results of *in vitro* studies on melanoma radiosensitivity suggest high intrinsic capacity of melanoma cells for repair of sublethal DNA damages caused by photon beam radiotherapy.^11^–^14^ This particular characteristic of melanoma cells is graphically presented by a distinctly broad shoulder in the low-dose portion of the logarithmic cell survival curve (Figure 1).^13^ However, variations in the cellular radiosensitivity recognized *in vitro* and in clinic imposed other factors to add to the observed heterogeneity among treated tumors.^10^,^12^ There are several candidates, *i.e.* intra-tumor variability (clonogenic subpopulations with different radiosensitivity, variations in tendency to apoptosis); tumor physiological factors (the existence of hypoxic fraction and/or differences in reoxygenation capacity of tumor clonogens, the intracellular level of glutathione – scavenger of free radicals responsible for DNA damage); tumor cell kinetics (different propensity to cell cycle disruption); and host-related factors (immune competence of the patient).^9^–^12^ According to the results of *in vitro* studies, sublethal irradiation doses increase the risk of metastases, possibly due to increased hypoxic fraction and hypoxia-induced up-regulation of urokinase-type plasminogen activator receptor in regrowing primary tumors.^15^

Theoretically, the ability of melanoma cell to overcome sublethal DNA injuries caused by irradiation suggests that, clinically, melanoma should be more sensitive to large doses per fraction (hypofractionation) than to lower fraction doses (hyperfractionation).^14^ This concept is mirrored in a low value of the ratio of the parameters α and β in the linear-quadratic model, a determinant of the shape (or bendiness) of survival curve in the model and an indicator used to quantify the fractionation sensitivity of tissues. As derived from clinical data, the α/β ratio for cutaneous melanoma ranges from 0.6 Gy to 2.5 Gy and is characterized by wide confidence intervals, implying large variations in the sensitivity of individual tumors to radiotherapy.^7^,^8^ Also, wide range of values of the α/β ratio resulted from calculations in preclinical studies.^16^

Despite seemingly firm theoretical arguments, clinical data on optimal fractionation pattern are not equivocal and no consensus was accepted on the best radiation regimen. This issue is further complicated by an increased probability of morbidity from late reacting normal tissue injury when using hypofractionated regimens.^13^,^14^ Whereas good arguments for the use of fraction doses of ≥4 Gy were provided by several retrospective studies (for review see Bello *and* Ang^17^), the results of the only prospective randomized trial addressing the issue of low versus high fraction doses neglected the expected advantage of hypofractionation. In RTOG 83-05, 137 patients with measurable lesions were randomized between 20 × 2.5 Gy in 26–28 days and 4 x 8 Gy in 21 days.^18^ No differences in local control, either complete or partial, were reported between the two arms; unfortunately, no data on the duration of responses were provided from this trial.^18^ On adjuvant setting, a retrospective comparison of conventional and hypofractionated regimens^19^–^22^ and reported in-field relapse rates from rare retrospective^23^ or prospective^24^ series implementing more conventional fraction doses (*i.e.* 1.7–2.4 Gy) support this observation.

On the other hand, prospective randomized comparison of different hypofractionation schedules (3 × 9 Gy versus 5 × 8 Gy, 2 fractions per week) in recurrent or metastatic melanoma resulted in virtually identical durable complete response rates of 65% and 72%, respectively, in the two arms of the trial.^25^ In another randomized study, after adding hyperthermia as an adjuvant to radiotherapy (3 × 8–9 Gy in 8 days) for macroscopic lymph node and skin disease, multivariate analysis with either complete response or 2-year local control rates as an endpoint showed that tumor dose, on the top of additional hyperthermia and tumor size, was an independent prognostic variable.^26^

## Indications for radiotherapy

Considering the treatment intent and the time point at which radiotherapy is to be introduced into melanoma management, indications for irradiation can be divided into four groups: upfront radiotherapy (as the main treatment modality, replacing surgery); adjuvant radiotherapy (after surgery), elective and palliative radiotherapy.

### Radiotherapy as primary therapy

Radiotherapy is rarely used as a primary treatment modality instead of surgery which is the curative treatment of choice for all types of primary melanoma lesions. Poor performance status of the patient with severe comorbidities or refusal of proposed surgery are potential but less plausible motives in clinic for replacing surgery with radiotherapy.

More frequent indication for upfront radiotherapy is lentigo maligna melanoma (LMM). Particularly when LMM is extensive and located on the face of elderly patient, radiotherapy is a good alternative to surgery. In three larger series with a total of 107 patients, 3 local recurrences were observed 13–44 months after radiotherapy (85 lesions) or combination of surgical excision of the nodular part followed by irradiation of the lentiginous part of the lesion (22 lesions).^27^–^29^ Time to complete regression of the lesion after irradiation took up to 24 months. Regional node metastases developed in 3 patients 6, 8 and 18 months after therapy, respectively, whereas in one patient, pulmonary metastases occurred 44 months after treatment. All theses patients had their primaries controlled.^27^–^29^ Thus, whenever surgery attempting to achieve clear margins would result in excessive mutilation, either cosmetic or functional, or in elderly patients, it should be replaced with radiotherapy, which is effective and has curative potential in LMM. Because the incidence of regional metastases is extremely low, no elective irradiation of regional lymphatics is required.

Primary curative radiotherapy should be attempted also in localized inoperable mucosal melanoma (MM) where it is considered the most effective treatment modality. After the extensive literature review, Krengli *et al.* summarized their analyses in a way that high local control rates, over 70%, can be achieved with radiotherapy alone in MM, which could be – taking into account some preliminary results – further improved by utilizing high-linear energy transfer (LET) radiation.^30^ Primary tumor area only should be included into the irradiation field in clinically N0 disease as it is unlikely that elective nodal treatment affects the overall course of the disease.^30^,^31^ The exception might be the oral cavity primaries with higher regional failure rates.^32^

### Adjuvant radiotherapy – primary melanoma

After excision of primary lesion, the decision about the use of postoperative radiotherapy is dictated by the risk estimate for recurrence, treatment related side-effects and the possibility for successful salvage when recurrence occurred. Because of superficial nature of the target tissue(s), the risk for serious complications after local radiotherapy is low.

Factors that adversely influence local control after wide excision alone are close or positive margins, early and/or multiple recurrences, extensive satellitosis, desmoplasia or neurotropism, and MM primaries. The incidence of local recurrence when tumor satellites are noted histologically was reported to be 12–14%^33^,^34^, and in desmoplastic tumors, as high as 11–48%.^35^–^38^ In the latter case, it appears that local recurrence may be related to the presence of neutropism^38^ and to inadequate surgical margins^36^–^39^, which could be of importance for lesions arising in anatomically critical regions of the head and neck. In high-risk clinical situations, postoperative radiotherapy has a potential to reduce the risk of local recurrence significantly.^37^,^40^–^42^ In MM, a number of retrospective studies suggest that postoperative radiotherapy yields better outcome, although it has no influence on survival. Combined approach is currently recommended after non-radical surgery, but seems to improve local control also after excision of large primary tumors, especially those in sinonasal localization, and those with perineural invasion.^30^

### Adjuvant radiotherapy – regional lymphatic metastases

After dissection of regional lymph nodes, radiotherapy adds significantly to an improved control in the operative bed. Only two randomized controlled trials were conducted to clarify this issue. The first was carried out in the 1970s with small sample size (56 patients) using an unusual regimen (split course, 50 Gy total and 1.78 Gy daily mid-plane dose, one field was treated daily) and was found inconclusive.^43^ Only recently, the results of the intergroup multicenter randomized trial (ANZMTG 01.02/TROG 02.01) were published (in an abstract form, Henderson *et al.*^44^). After lymphadenectomy for isolated regional recurrence of melanoma, 250 patients considered to be at high risk (>25%) of in-field recurrence were randomized into radiotherapy group (126 patients) and control group (127 patients); 227 patient were available for analysis. After a median follow up of 27 months, a statistically significant improvement in lymph node field control was observed with radiotherapy (hazard ratio 1.77, 95% confidence interval 1.02–3.08, P=0.041), but not also in median survival times (P=0.14).^44^

Thus far, identification of factors increasing the risk for regional recurrence after lymphadenectomy and recommendations for adjuvant irradiation were based on retrospective analyses or rare non-randomized prospective studies. In high-risk setting, the rates of relapse in nodal basin could reach 50% or even more after surgery alone. The factors contributing to an increased recurrence in surgical field are the presence of residual disease after surgery, extracapsular tumor extension, nodes measuring ≥3 cm in the largest diameter, multiple nodal involvement or recurrence after previous lymph node dissection (and RT was not used at that time) (Table 1).^22^,^45^–^61^ The criteria for using adjuvant irradiation vary slightly among different nodal basins, reflecting various potential outcomes for distinctive anatomical body region. In recent ANZMTG/TROG randomized trial, the high-risk features (in addition to non-radical surgery and recurrent disease) were as follows: ≥1 parotid, ≥2 cervical or axillary or ≥3 groin nodes; extracapsular spread of tumor; maximum metastatic node diameter ≥3 cm in neck and axilla or ≥4 cm in the groin.^44^

Comparison of studies using surgery alone or surgery plus radiotherapy provides a strong argument for the effectiveness of adjuvant irradiation when adverse prognostic factors are found at histopathological examination of resected specimen. In-field tumor control is roughly 90% in adjuvantly irradiated patients using either conventional or hypofractionated schedules (Table 2).^21^–^24^,^41^,^44^–^49^,^51^–^70^ However, in these studies, only the patients with less favorable disease characteristics were referred to radiotherapy; thus, the existing selection bias should be aware of when comparing the results between these two groups of studies. Finally, while matching the results of studies simultaneously reporting on the outcome in surgically and postoperatively irradiated patients, it seems that adjuvant radiotherapy compensates effectively for the negative impact of adverse histopathological features to the disease control in the dissected nodal basin (Table 3).^22^,^44^,^47^,^51^,^61^,^69^,^71^ No effect of postoperative irradiation on survival was observed in these studies. To the contrary, Agrawal *et al.* recently reported that adjuvant radiotherapy also could have a positive impact on melanoma specific survival.^61^

Owing to the increased probability of serious treatment-related side effects after adding radiotherapy to surgery, particularly lymphedema, and due to high likelihood of distant metastases in the patients with extensive lymph node involvement and no survival advantage for the adjuvantly irradiated patients, the question has been raised on the meaning of adjuvant use of radiotherapy. In view of these obstacles, the primary goal of postoperative irradiation must be emphasized, *i.e.* to prevent the uncontrolled regional recurrences with local destruction and associated infection (with secretion and stench), hemorrhages, edema, disfigurement or pain, which produce considerable morbidity that significantly reduces the quality of patient’s life.

The probability of systemic dissemination, which is the most powerful predictor of the risk of dying due to the disease, seems to be associated with the number of involved nodes.^22^,^70^ Thus, it sounds reasonable to use as cut point a certain number of involved nodes at which the risk of distant failure is that high that regional radiotherapy should not be delivered, despite its proven effectiveness in controlling regional disease. A reasonable number might be between 10–15 nodes, at which point the risk of distant metastasis reaches 70%.^22^,^70^

When a comprehensive nodal resection is not done and only local excision of palpable node(s) is performed instead, either due to significant medical comorbidities or patient’s refusal of more extensive surgical procedure, radiotherapy seems to have a potential to compensate for this deficiency. In a series of 36 patients with parotid or cervical node metastases from melanoma treated with local excision of palpable nodal disease and postoperative radiotherapy (to the primary site – if known, the site of nodal excision and the undissected ipsilateral neck), the disease, after the median follow up of 5.3 years, recurred within the regional basin in two patients only and at distant sites in 14 patients.^72^ In this setting, it seems unlikely that a comprehensive surgical dissection would improve the regional control, but observation only would place the patient at unnecessary risk of regional recurrence.

### Elective radiotherapy – regional lymphatic metastases

Elective neck irradiation is a viable treatment option for the patients at risk for nodal micrometastases who are not candidates for sentinel lymph node biopsy.^62^,^73^ In a retrospective series of 157 patients with high risk cutaneous melanoma of the head and neck for lymph node involvement (stages I or II), elective regional radiotherapy was found effective and safe treatment option. After a median follow up of 68 months, the disease recurred in the neck lymph nodes in 15 patients and distantly in 57 patients.^73^ However, in the sentinel lymph node dissection era, this particular indication is less relevant.

### Palliative radiotherapy

The primary goal of palliative radiotherapy is to reduce signs and symptoms related to the disease and improve quality of patients’ life; eventual prolongation of her/his life is in the second plane.

Palliative RT is to be introduced whenever surgery is not possible (*i.e.* technically unresectable tumors, poor general condition of the patient) or is deemed ineffective (*i.e.* multiple metastases, particularly when occurring in different organs). In general, all types of metastases or metastatic sites can be irradiated, including cutaneous, lymphatic, brain, bone, and visceral lesions. The effectiveness of radiotherapy in palliative setting is primarily dependent on tumor burden and site. According to the results of *in vitro* studies, cells from metastatic lesions are more radioresistant than those from primary tumors.^74^

Whereas more than 85% complete response rate could be expected after irradiation of small-size (*i.e.* ≤1 cm in diameter) cutaneous lesions, the frequency of complete response is less than 30% in tumors of 5 cm in diameter or larger.^7^ Single-shot or fractionated radiotherapy of bone metastases results in complete or partial pain relief at one month after the completed therapy in more than 65% of cases.^75^ In the case of impending or known pathologic fracture, a combination of adjuvant irradiation that follows upfront surgical intervention resulted in immediate pain relief and prolongation of disease-free interval. Surgical treatment of existing bone fracture is indicated when the expected survival exceeds 6 weeks and the patient’s condition permits operation, when no greater benefit from nonoperative treatment is expected, when internal stability can be obtained and when early mobilization is possible. The criterion for choosing between radiotherapy and combined treatment approach when impending fracture is diagnosed is metastasis in weight-bearing bones of a diameter >2–3 cm or with cortical destruction of >50%.^75^

**FIGURE 2:**
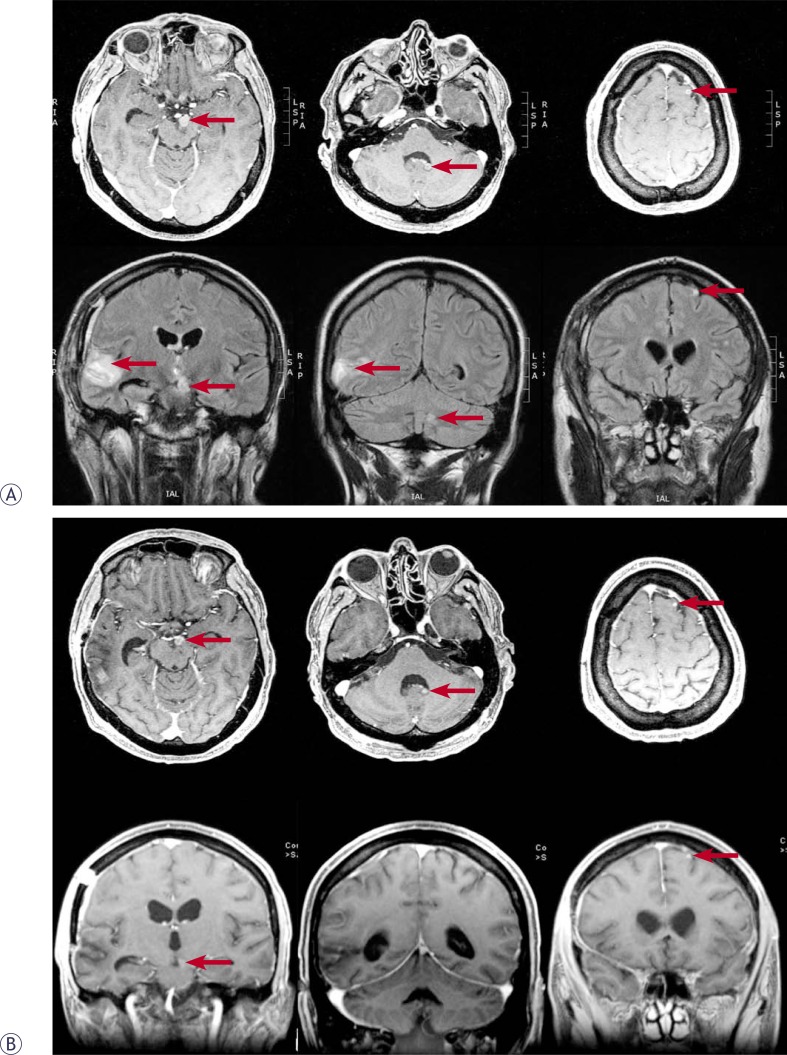
Stereotactic radiosurgery. This radiotherapy technique is characterized with maximal accuracy and is used for focal irradiation of small brain lesions (usually up to 3 tumors of 3.5 cm maximal diameter). After rigid fixation of the head with specific frame, several small beams coming from various directions are focused on one spot inside of the target, creating a steep dose gradient on periphery of the target. Tumor doses in the range of 16–25 Gy are prescribed on 80% isodose encompassing the lesion, whereas 1–2 mm from the edge of the target, the dose drops to 20–30% of its prescribed value. Local control is in the range of 90% and the prevailing cause of death is progression of extracranial disease. In January 2009, a 59-year-old male with melanoma, diagnosed 4 years earlier, presented with 4 metastatic lesions in the brain. On PET-CT, two additional metastases were identified elsewhere in the body, occupying the third lumbar vertebra and the musculature of the posterior abdominal wall. T1-weighted post-contrast MRIs revealed a lesion of 30x20 mm (long arrow) in the right temporoparietal region, a smaller one in the left half of the pons (short arrow), a 6 mm lesion in the left frontal lobe (thick arrow) and a 7 mm lesion in the left cerebellar hemisphere (arrowhead) (Figure 2A). The patient was treated with surgical resection of the large temporoparietal metastasis, whole brain irradiation (10 × 3 Gy), temozolamide and stereotactic radiosurgery of other three (smaller) brain metastases with the irradiation doses to 80% isodose of 20 Gy (the lesion in the frontal lobe) and 18 Gy (the lesions in the pons and cerebellum). Four months after the procedure, the size of all three irradiated tumors was reduced and no new lesion was identified in the brain (Figure 2B). In September 2009, disease progression was recorded after detecting a metastasis in the spinal cord which was treated with surgery, postoperative irradiation and chemotherapy. No progression of treated brain metastases occurred so far (January 2010, 11 months after stereotactic radiosurgery).

Patients with brain metastases are usually referred to whole brain radiotherapy (WBRT), whenever their number or location excludes surgical intervention or stereotactic radiotherapy. In combination with corticosteroids, WBRT resulted in life prolongation for uninspiring 1–2 months^76^, whereas an improvement of performance status, at least temporary, could be expected in 60–70% of patients.^17^ In a recent report on 686 melanoma patients with brain involvement, supportive carealone resulted in a median survival of only 2.1 months, WBRT 3.4 months, neurosurgery 9.7 months and combination of surgical resection and WBRT 8.9 months.^77^ For patients with lower number of metastatic lesions (usually ≤3) and of maximal diameter between 2.5–3 cm, stereotactic radiosurgery, either with linear accelerator or gamma-knife-based, represents a comparative alternative to surgery (Figure 2).^78^ In this clinical scenario, local control in the range of 90% with sporadic long-term survivorships can be expected, whereas in the majority of patients treated with stereotactic irradiation the prevailing cause of death is progression of extracranial disease.^77^,^78^ Recently, as no difference in local control or survival was found when WBRT and stereotactic radiosurgery versus surgery plus WBRT and a boost were compared, the less invasive of the two combinations, WBRT and stereotactic irradiation, was recommended as a treatment of choice for the patients with one or two brain metastases.^79^

In metastases causing spinal cord compression, radiotherapy can be used as a single modality (in conjunction with high dose corticosteroids) or in combination with surgery to reverse neurological impairment or to prevent further loss of motor functions.^80^ The decision on the use of upfront surgery versus radiotherapy alone depends on the assessment of neurological deficit, mechanical instability, radioresponsiveness and extent of malignant disease, patient’s performance status and comorbidities. Combined treatment offers good chance for pain relief and restoration of affected neurological functions as well as delay in tumor regrowth and prolongation of symptoms-free period.^80^,^81^

## Radiotherapy regimens and techniques

As the best radiotherapy regimen for melanoma remains undetermined, fractionation pattern should be in line with treatment intent and adapted to treated patient: *i.e.*, anatomical localization and extent of radiation volume/target, life expectancy and convenience for the patient, taking into account her/his performance status and preferences. All existing radiotherapy armamentarium can be used when irradiating melanoma, from simple kilovolt-age machines or telecobalts to sophisticated linear accelerators, tomotherapy units or cyber-knife.

At the Institute of Oncology Ljubljana, in melanoma patients irradiated with curative intent, the choice of fractionation pattern and total dose is governed mainly by the region to be irradiated. If there is no particular risk for lymphedema, *e.g.* targets on the trunk or neck region, higher fraction doses are used (4–6/fx Gy), although, owing to the risk of subcutaneous fibrosis particularly on the neck, lower fraction doses are sometimes preferred. In other clinical scenarios (axilla, groin), more conventionally fractionated radiotherapy regimens are implemented (1.8–2.5 Gy/fx). In palliative radiotherapy, smaller number of higher daily doses is usually employed (4–8 Gy/fx). The complexity of treatment plans, including the number of beams implemented, beam shaping and irradiation techniques (simple 2D, 3D-conformal, intensity modulated, image-guided) is also adjusted to the treated region and treatment intent.

For macroscopic disease, curative dose should be in a range of 66–70 Gy (equivalent dose, *i.e.* when conventionally fractionated with 2 Gy/day and 5 fractions/week, α/β = 2 Gy; Jones *et al.*^14^). Radiotherapy dose prescribed postoperatively to the operated side of the neck should be in the range of ≥60 Gy^22^,^41^,^47^,^61^–^63^,^68^,^70^,^71^, although a favorable outcome was also reported with lower doses.^21^–^24^ For irradiation of axillary and inguinal nodal basins, a total dose of 50–55 Gy, causing a tolerable profile of irradiation induced side-effects, is used as recommended.^24^,^44^ In palliative setting, the radiotherapy doses are usually lower (equivalent dose 24–50 Gy).

The stereotactic technique is a valuable option for clearly defined subset of patients with brain metastases. It based on rigid fixation of the head with specific frame, allowing more accurate positioning of the head (and tumor – target) in 3-dimensional space compared to non-stereotactic conditions. Several small beams coming from various directions are focused on one spot inside of the target, creating a steep dose gradient on periphery of the target. Tumor doses in the range of 16–25 Gy are prescribed on 80% isodose encompassing the lesion, whereas 1–2 mm from the edge of the target the dose drops to 20–30% of its prescribed value (Figure 2).

## Conclusions

Basic treatment modality in melanoma is surgery. However, whenever surgery is not radical or there are adverse prognostic factors identified on histopathological examination of resected tissue specimen, it needs to be supplemented. Also, in patients with unresectable disease or in those not being suitable for major surgery or who refuse proposed surgical intervention, other effective mode(s) of therapy need to be implemented. From this perspective, supported by clinical experiences and literature results, radiotherapy is a valuable option: it is effective and safe, in curative and palliative setting. However, the highest benefit in terms of best achievable disease control rates and, simultaneously, minimal treatment-related toxicity is obtainable when modern radiotherapy equipment and techniques are used and indications for irradiation are followed consistently, on patient-to patient basis.

## Figures and Tables

**TABLE 1:** Nodal field relapse rates (number of relapses/dissections) after therapeutic surgery according to adverse clinicopathological features negatively impacted disease control in dissected nodal basin

**Parameter**	**Nodal basin recurrence (%)**	**References**
**No. of involved nodes**		
1	9, 9, 25, 45	45,46,47,48
1–3	19, 14, 25^a^, 15, 24	47,49,50^a^,^51^,^52^
2–4	15, 10	45,46
≥4	17, 22, 20, 60, 53, 46^a^, 8, 37	45,46,47,48,49,50^a^,^51^,^52^
>10	33, 26, 63^a^, 47	45,46,50^a^,^52^
**Diameter of largest node**		
<3 cm vs. 3–6 cm vs. >6 cm	25 vs. 42 vs. 80^a^	50^a^
**Extracapsular tumor spread**		
No	15, 38, 23^a^, 9	45,48,50^a^,^51^
Yes	28, 54, 63^a^, 24, 43	45,48,50^a^,^51^,^52^
**Matted nodes**		
Yes	29, 44, 12	45,46,52
**Nodal basin**		
Parotid & neck	41,15, 19, 50, 43^a^, 14, 43, 50, 50	22,45,47,48,50^a^,^51^,^52^,^53^,^54^
Axilla	15, 60, 28^a^, 30, 14, 10	45,48,50^a^,^52^,^53^,^55^
Groin	17, 44, 18, 23, 19, 8, 34, 19, 34, 8	45,48,49,50^a^,^52^,^53^,^56^,^57^,^58^,^59^
**All nodal sites**	16, 52, 18, 30^a^, 28, 15, 34, 41	45,48,49,50^a^,^52^,^53^,^60^,^61^

a Actuarial nodal basin control rates at 10 years are reported.

**TABLE 2:** Therapeutic lymph node dissection in melanoma patients with or without adjuvant radiotherapy: comparison of nodal basin recurrence rates

**Surgery**			**Surgery plus radiotherapy**		
**Author, year^Ref.^**	**No. of pts.**	**Nodal basin recurrence (%)**	**Author, year^Ref.^**	**No. of pts.**	**Nodal basin recurrence (%)**
***Parotid & neck***			***Parotid & neck***		
Bayers, 1986^54^	28	50	Ang *et al*., 1994^62^	95	8
Calabro *et al*., 1989^45^	287	15	O’Brian *et al*., 1997^47^	45	7
O’Brian *et al*., 1997^47^	107	19	Shen *et al*., 2000^51^	21	14
Shen *et al.*, 2000^51^	196	14	Ballo *et al*., 2002^63^	160	8
Pidhorecky *et al*., 2001^52^	44	43	Strojan *et al*., 2010^22^	45	18
Strojan *et al*., 2010^22^	42	40	*Total*	366	10
*Total*	704	20			
***Axilla***			***Axilla***		
Bowsher *et al*., 1986^53^	22	14	Ballo *et al.,* 2002^64^	89	10
Calabro *et al*., 1989^45^	438	15	Beadle *et al*., 2009^65^	200	10
Pidhorecky *et al*., 2001^52^	116	30	*Total*	289	10
Kretschmer, *et al*., 2001^55^	63	10			
*Total*	639	17			
***Groin***			***Groin***		
Bowsher *et al*., 1986^53^	36	8	Ballo *et al*., 2004^66^	40	23
Kissin *et al.*, 1987^56^	44	34			
Calabro *et al*., 1989^45^	276	17			
Hughes *et al*., 2000^57^	132	19			
Pidhorecky *et al*., 2001^52^	93	19			
Kretschmer *et al*., 2001^58^	104	34			
Allan *et al*., 2008^59^	72	8			
*Total*	757	20			
***All sites***			***All sites***		
Bowsher *et al.*, 1986^53^	66	15	Burmeister *et al*., 1995^67^	26	12
Calabro *et al*., 1989^45^	1001	16	Corry *et al*., 1999^23^	42	21
Miller *et al*., 1992^49^	55	18	Stevens *et al*., 2000^68^	174^1^	11
Monsour *et al*., 1993^48^	48	52	Cooper *et al*., 2001^41^	40^1^	8
Pidhorecky *et al*., 2001^52^	253	28	Fuhrmann *et al*., 2001^69^	58	16
Mayer *et al*., 2002^60^	140	34	Chang *et al*., 2006^21^	54	12
Henderson *et al*., 2009^44^	108	31	Burmeister *et al*., 2006^24^	234	7
Agrawal *et al*., 2009^61^	106	41	Ballo *et al*., 2006^70^	466	9
*Total*	1777	23	Henderson *et al*., 2009^44^	123	18
			Agrawal *et al*., 2009^61^	509	10
			*Total*	1726	11

**TABLE 3:** Nodal basin control after surgery with or without adjuvant radiotherapy

**Author, year^Ref.^**	**No.**	**Nodal Basin**	**Risk factors (%)**			**FUP, median (mos.)**	**Nodal basin recurrence (%)**		**Survival, at 5 yrs. (%)**	
	
			**ECE**	**N_+_ ≥2**	**N_+_ >3**		**Absolute**	**Actuarial (at 5 yrs.)**	**Melanoma specific**	**Overall**
O’Brian *et al*., 1997^47^										
Surgery	107	Parotid & neck	20	43	9	56	19	40	35	n.r.
Surgery + XRT	45	Parotid & neck	49	67	24	38	7	17	40	n.r.
Shen *et al*,. 2000^51^										
Surgery	196	Parotid & neck	23	n.r.	27	32^a^	14	17	n.r.	32
Surgery + XRT	21	Parotid & neck	43	n.r.	48	n.r.	14	25	n.r.	n.r.
Fuhrmann *et al*., 2001^69^										
Surgery	58	All sites	n.r.	74	n.r.	n.r.	21	26	n.r.	25
Surgery + XRT	58	All sites	n.r.	74	n.r.	n.r.	16	22	n.r.	23
Moncrieff *et al*., 2008^71^										
Surgery	587	Parotid & neck	n.r.	n.r.	n.r.	35	n.r.	6^b^	n.r.	n.r.
Surgery + XRT	129	Parotid & neck	n.r.	n.r.	n.r.	35	n.r.	10^b^	n.r.	n.r.
Henderson *et al.*, 2009^44^										
Surgery	108	All sites	All patients at high risk for regional recurrence			27	31	n.r.	n.r.	n.r.
Surgery + XRT	109	All sites				27	18	n.r.	n.r.	n.r.
Agrawal *et al*., 2009^61^										
Surgery	106	All sites	All patients at high risk for regional recurrence			60	41	48	30	n.r.
Surgery + XRT	509	All sites				60	10	13	51	n.r.
Strojan *et al*., 2010^22^										
Surgery	42	Parotid & neck	21	38	n.r.	25	40	44^a^	n.r.	58^a^
Surgery + XRT	45	Parotid & neck	44	64	n.r.	25	18	22^a^	n.r.	51^a^

ECE – Extracapsular extension of tumor; N_+_ - Number of positive nodes; FUP – Follow-up; XRT – Radiotherapy; n.r. – Not reported.

a At 2 years.

b At 6 years.
